# The Double-Edged Effects of Substance P in the Pathology of Alzheimer’s Disease

**DOI:** 10.14336/AD.2024.0960

**Published:** 2025-10-15

**Authors:** Zihan Lin, Shuyao Yu, Yuling Yang, Hongyu Mu, Yidan Hu, Weihua Yu, Yang Lü

**Affiliations:** ^1^Department of Geriatrics, The First Affiliated Hospital of Chongqing Medical University, Chongqing, China.; ^2^International Medical College of Chongqing Medical University, Chongqing, China.; ^3^Institutes of Neuroscience, Chongqing Medical University, Chongqing, China

**Keywords:** Alzheimer's disease, substance P, neuroinflammation, neuroprotection

## Abstract

Alzheimer's disease is an irreversible neurodegenerative disease that manifests clinically as memory loss and so on. Neuroinflammation plays an important role in Alzheimer's disease. Meanwhile, widely distributed throughout the central nervous system, substance P exhibits important pro-inflammatory properties. The level of substance P is found to correlate with the course of Alzheimer's disease. Substance P can modulate the protein hydrolysis of amyloid precursor protein, the voltage-gated potassium channel, and the protein hydrolysis of this channel, exerting neuroprotective effects. At the same time, substance P can also exert damaging effects by mediating neuroinflammation, inhibiting cellular autophagy, activating mast cells, acting on leukocytes and altering blood-brain barrier permeability. Based on the complex manifestations of substance P in Alzheimer's disease, this review discusses both protective and damaging mechanisms, and plausible explanations for the double-edged effect of substance P, providing an outlook for future research focusing on substance P and Alzheimer's disease.

## 1. Introduction

Alzheimer's disease (AD) is one of the most common degenerative diseases of the nervous system, which is primarily characterized by the gradual decline in cognitive functions, accounting for 50-75% of dementia [1]. More than 30 million people worldwide have AD [2]; however, so far, there is no effective therapy due to the incomplete understanding of AD etiology and pathogenesis.

The primary clinical manifestations of AD include memory loss, thinking obstacles and language difficulties [3]. The main pathological features are the appearance of amyloid plaques and neurofibrillary tangles (NFTs) in the cerebral cortex. In addition, dystrophic neural synapses, astrogliosis, and microglia activation were also observed in AD. Downstream consequences of these pathological processes include neurodegeneration and macroscopic atrophy of brain tissue caused by synaptic and neuronal loss [2]. Currently, aside from cholinesterase inhibitors and memantine, there are not many drugs effective for AD. While they are available to improve memory and cognition in patients with AD, they cannot alter life expectancy or the overall progression of AD. Thus, there is an urgent need to develop novel and effective approaches for the early diagnosis and successful treatment of AD disease.

Substance P (SP) is an 11-aa neuropeptide, a member of the tachykinin family [4]. Being the most prevalent tachykinin in the brain, SP exhibits widespread distribution within the central nervous system (CNS).

There has been increasing evidence suggesting that SP plays an important role in AD in different aspects. In both human AD patients and animal AD models, there has been a noticeable decrease in the level of SP within the hippocampus which is critically involved in the formation of memory and plays a significant role in the development of AD pathology. SP has also been observed to exhibit neurotrophic and neuroprotective effects in both in vitro and in vivo studies [4], with strongly proven memory improvement functions in several animal experiments [5]. However, its negative effects are also obvious, as SP mediates neuroinflammation, inhibiting cellular autophagy [4] as well as activating different signaling pathways including NF-κB pathways [2].

SP, by binding to and activating NK-1R, plays a role in controlling various biological functions. It has participated in many signaling pathways including both neuroprotective and injurious ones. The neuroprotective role of SP against amyloid β-protein (Aβ) could be related to its ability to modulate voltage-gated potassium (Kv) channel currents. In addition, SP is able to stimulate non-amyloidogenic processing, thus reducing the possibility of generation of toxic Aβ in the brain. Meanwhile, it has been reported that the interaction between SP and NK-1R can promote the production of proinflammatory cytokines via NF-κB pathways, which plays a significant role in AD neuroinflammation progression [6, 7].

It is also recommended that considering the fluctuation of SP in early-staged and late-onset AD, SP should be recognized as a biomarker of AD, because it may symbolize the compensatory mechanism against the pathological deposit of Aβ, which is proved by the observed positive correlation between the level of SP and Aβ [5]. Meanwhile, the activity of neuropeptidases that metabolize SP also presents changes in AD patients, which means the change in the activity of SP does take place in AD patients and suggests great significance [8].

However, changes of SP level during the progression of AD are complex, with large differences according to different factors. SP expression also varies in different brain regions [5]. Meanwhile, SP is positively correlated with the level of Aβ_1-42_, the biomarker of the disease status in AD patients [8]. Elevated cerebrospinal fluid (CSF) SP levels may play a compensatory role in protecting patients from pathological exposure.

**Figure 1.**
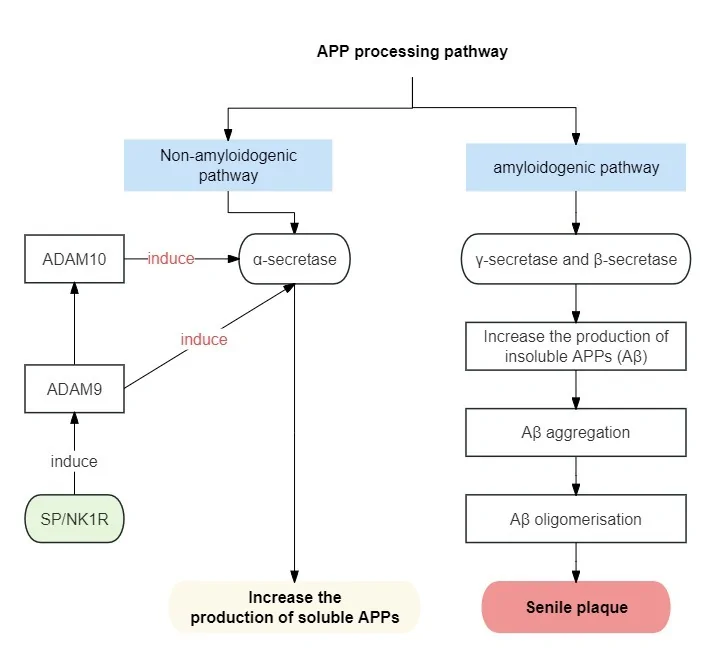
**APP processing pathways [11]**. This figure illustrates the APP processing pathways including the non-amyloidogenic pathway and amyloidogenic pathway. On the right side, the amyloidogenic pathway is depicted, involving the enzymes γ-secretase and β-secretase. These enzymes sequentially process APP in a way that increases the production of insoluble APPs known as Aβ. This pathway further leads to the aggregation of Aβ, followed by the oligomerization of Aβ, eventually forming senile plaques which are characteristic of Alzheimer's disease. Within the non-amyloidogenic pathway, proteolytic cleavage of Aβ peptide sequence happens by interactions with α-secretase, producing large amounts of soluble APPs. By binding to NK1 receptor, SP induces the overexpression of ADAM9 which further induces ADAM10. Both proteins can function as α-secretase, therefore, leading to increased production of sAPPs.

Therefore, by exploring the potential role of SP in AD, there may be great opportunities to provide better management in AD patients. Based on the complicated manifestations of SP and its extensive role in AD patients, this review will address both protective and damaging mechanisms separately and therefore summarize its double-edged effects. Through literature research, we have discovered a substance closely related to the development of Alzheimer's disease - substance P. The role of SP is extensive, and its effects on AD vary in different studies. Our review has summarized the double-edged sword effects of SP in AD (Table 1).

**Table 1:** SP in AD: The Summary of Double-Edged Sword Effects.

	Target Site	Signaling Pathway
**The Protective Effects**		
**Promotion of non-amyloidogenic pathway**	α-secretase (ADAM9, ADAM10)	By activating ADAM9 and ADAM10, increasing the secretion of sAPPα, promoting the processing of APP, and reducing the production of Aβ
**Activation of Akt pathway**	NK1R, Akt	SP activates NK1R to phosphorylate Akt, inhibits the activity of Kv4 potassium channels induced by Aβ, increases the concentration of p-Akt, thereby suppressing Aβ-induced cell apoptosis
**Co-aggregation to inhibit Aβ toxicity**	Co-aggregation of SP and Aβ	SP co-aggregates with Aβ, reduces Aβ toxicity, and exerts neuroprotective effects
Activation of neuroinflammation	Clear infection	Down-regulating IL-6 increases M2 and decreases M1, promoting regeneration and repair of neuron
	**Microglia**	JAK/STAT pathway, activated by IL-6, promotes nerve regeneration
	**Cytolines**	mTOR pathway, activated by Ras, promotes nerve regeneration
**The Injurious Effects**		
**Activation of microglia**	NK-1R on the surface of microglia	Activation of the mTOR and NF-κB pathways, leading to pro-inflammatory effects and dysregulation of autophagy
**Activation of mast cells**	NK-1R on the surface of mast cells	SP stimulation induces mast cell degranulation, releasing pro-inflammatory cytokines and neuroactive substances such as IL-1β, IL-6, TNF-α, contributing to neuroinflammation
**Activation of the HPA axis**	Not specified	SP upregulates CRHR-1 leading to increased production of IL-6, TNF-α, and VEGF, contributing to neuroinflammation
**Acting on leukocytes**	Not specified	SP modulates leukocyte activation, immune function, T cell subset differentiation, and adaptive immune activation
**Increasing blood-brain barrier permeability**	Not specified	SP increases BBB permeability by directly affecting angiogenesis and disrupting tight junction proteins, exacerbating neuroinflammation and neurodegeneration

SP, substance P; AD, Alzheimer’s disease; ADAM, a disintegrin and metalloproteases; sAPPα, soluble amyloid precursor protein; APP, amyloid precursor protein; Aβ, amyloid β; NK1R, Neurokinin 1 receptor; Akt, serine/threonine kinase; Kv channel, voltage-gated potassium channel; IL-6, interleukin-6; M1 & M2, status of microglia; JAK/STAT, Janus kinase/Signal Transducers and Activators of Transcription; mTOR, mammalian target of rapamycin; NF-κB, nuclear factor kappa B; IL-1β, interleukin-1β; CRHR, corticotropin-releasing hormone receptor; VEGF, vascular endothelial growth factor; TNF-α, tumor necrosis factor-α; IL-1β, interleukin-1β; BBB, blood brain barrier.

## 2. Protective effects

### 2.1 Neuroprotection associated with Aβ

#### 2.1.1 Non-amyloidogenic pathway

Research conducted in vivo has demonstrated that within the mature brain, Aβ serves as a notable neurotoxin, but its harmful impacts can be alleviated by SP [9]. It is established that the abnormal extracellular deposition of neurotoxic amyloid peptides deriving from the proteolytic process of amyloid precursor protein (APP) is one of the characteristics of AD. This process contains two pathways which include non-amyloidogenic and amyloidogenic.

Within the amyloidogenic pathway, APP undergoes sequential cleavage by β-secretase and γ-secretase enzymes, resulting in subsequent neuronal apoptosis and the generation of Aβ, which is a key pathological feature of AD.

In contrast, in the non-amyloidogenic pathway, instead of β-secretase and γ-secretase enzymes, α-secretase plays the crucial role in facilitating the proteolytic processing of APP, resulting in the formation of a type of large extracellular soluble amyloid precursor protein (sAPPα). sAPPα can exert neuroprotective and trophic properties by leading to cleavages within the Aβ peptide sequence, reducing the deposition of Aβ. ADAM9, 10 and 17 of the ADAM family of multidomain transmembrane proteins have already been proven to function as α-secretases. sAPPα secretion can be increased by SP through the activation of ADAM9. It has been shown that SP can increase the expression of the proenzyme form of ADAM9, and then facilitate its maturation by promoting the cleavage of the prodomain motif, significantly modifying the functions of ADAM9 [10]. Another way for SP to indirectly increase the amount of sAPPα is via ADAM10. There have been theories that ADAM9 may have a role in the proteolytic maturation of ADAM10. Inhibition of ADAM9 protease activity reduces the shedding of ADAM10, leading to an increase in membrane-bound ADAM10. This, in turn, promoted higher levels of soluble APP through α-secretase, resulting in decreased Aβ levels. This suggests SP can increase α-secretase activity and regulate APP processing and Aβ production, thus greatly protecting neurons from apoptosis (Fig. 1) [10].

#### 2.1.2 SP co-aggregate with Aβ

SP has been proven to be able to co-aggregate with Aβ, which therefore can reduce Aβ’s toxicity, performing neuroprotective effects.

In the study by Liu et al., it was found that SP and Aβ (25-35) have similar primary structures, but the amylofibrils SP forms are non-toxic [12]. However, although the primary structures of SP and Aβ (25-35) are similar, both having a C-terminus composed of Phe, an aromatic or aliphatic residue (X), Gly, Leu and Met-NH2, their secondary structures are different [13]. The hexamer structure of Aβ (25-35) has been identified as cylindric, and strongly associated with its toxicity. Even though SP has a significant structural homology with Aβ (25-35), it does not form hexamers with the β-sheet structure like Aβ (25-35), which is the main contributor to its toxicity [12]. In vitro experiments of Flashner’s team that mix Aβ with SP in a ratio of 1:1 and incubate the mixture at 37°found that the mixed fibrils grown in the presence of SP exhibited reduced Thioflavin fluorescence, suggesting that SP might be able to co-assemble with Aβ, thus reduce Aβ toxicity [14]. Liu’s team also discovered that SP could reduce Aβ toxicity. According to their experiments, SP can promote Aβ(25-35) oligomerisation but also precipitate Aβ(25-35) oligomers, thereby reducing their concentration in the solution phase [12]. The dissolved Aβ oligomers are considered as a key factor in AD pathogenesis, as demonstrated in several studies that instead of the deposited Aβ, the dissolved Aβ oligomers can cross the cytoplasmic membrane and induce intracellular toxicity, and that oligomers are soluble. Toxicity in AD is primarily associated with dissolved oligomers because of their ability to pass between cells and trigger cytotoxicity. In contrast, deposited Aβ plaques are more of a pathological marker, not directly causing the same degree of toxicity. SP promotes the aggregation of Aβ monomers to form oligomers, helps these oligomers to precipitate out of solution, and thus plays a protective role by reducing toxic soluble oligomers in solution.

In summary, although SP and Aβ (25-35) share structural similarities, their different secondary structures explain why SP is non-toxic. SP also has the ability to co-aggregate with Aβ and reduce its toxicity, indicating the potential neuroprotective effects of SP.

### 2.2 Activation of neuroinflammation

SP can normally activate the neuroinflammatory response which serves as an inherent defense mechanism of the body in response to stimuli to protect normal brain structures and physiological function from infection and injury [15]. In neurodegenerative diseases, neuroinflammation can clear infection in the early stages to control disease progression. It has also been shown that neuroinflammation can, to some extent, facilitate the repair of damaged neurons and promote regeneration [16]. Microglia is one of the most important CNS immune cells, mainly acting as the main phagocytic cell in CNS. Although the phagocytic ability of microglia is limited, to some degree, they can still exert some beneficial effects in neuroinflammation via clearing detrimental substances in the brain parenchyma [17]. It is known that both CNS microglia and peripheral macrophages can be categorized into 2 general ranks, M1 and M2. While M1 is related to proinflammatory effects, M2 is more likely to exert protective influences, which means whether microglia and macrophages accelerate inflammation or facilitate regeneration depends on whether M1 or M2 outperforms. And this balance can be under many other regulations, such as cytokines. It has been discovered that down-regulating interleukin-6 (IL-6) pathways can contribute to an M2 increase, accompanied by an M1 decrease, which means in this way regeneration and repair are more in favor [16].

Cytokines can also play protective roles in many neurodegenerative cases. Early in 2000, animal models pre-treated with IL-1 were found to present descended neurological impairments [18]. A possible elucidation of this phenomenon is that IL-1 can induce the expression of growth factors with neuroprotective effects, such as insulin-like growth factor [17]. There are also some signaling pathways that play significant parts in AD pathology showing neuro-regeneration effects. Janus Kinase/Signal Transducers and Activators of Transcription (JAK/STAT) pathway activated by IL-6 and mammalian target of rapamycin (mTOR) pathways activated by Ras have been observed to have positive impacts in nerve regeneration [19].

Of note, both microglia and cytokines are connected very closely with SP; and SP can modulate a variety of their activities in AD pathology, which will be discussed in detail in this review later. There are a lot of interactions existing among these players, whereas the most impressive thing is that in contrast with the well-known roles SP plays, its regulation and effects in neuroprotection are unneglectable as well.

### 2.3 Akt pathway

The ability of SP to protect tissues from damage by anti-apoptosis has been demonstrated in some tissues. Chen and his team have directly shown that SP treatment can significantly decrease the apoptosis level in injured cardiomyocytes intervened by doxorubicin [20].

SP can activate serine/threonine kinase (Akt) via Neurokinin-1 Receptor (NK1R) to phosphorylate it, and Akt is considered to be a signaling molecule for cell growth and differentiation that can inhibit apoptosis Early from 2006, there has been literature demonstrating the anti-apoptosis effects exerted by Akt signaling. Akt signaling was reported to inhibit apoptosis by inhibiting apoptosis-related proteins such as BCL-2 like protein 11 and Caspase 9 [21]. The antiapoptotic effect of SP is mediated by the Akt pathway; while SP can activate Akt via NK-1R, which is a crucial signaling involved in cellular growth and apoptosis [22]. Previous evidence has shown that when cerebellar granule cells (CGCs) incubated by Aβ were treated with SP, the survival rate of CGCs was significantly raised, indicating the anti-apoptosis influence of SP (Amadoro et al., 2007). This suggests that Aβ can promote apoptosis followed by neurotoxic effects by reducing p-Akt concentrations, and the protective influence of SP in the CNS against Aβ-induced toxicity is mediated by the Akt pathway [22].

## 3. Injurious effects

### 3.1 Activation of microglia

Microglia are the main innate immune cells in CNS, which are essential in the homeostasis of CNS [23]. Genome-wide association studies (GWAS) have identified more than 20 AD-associated genetic variations in humans, and most of them are genes associated with the immune responses and phagocytosis of microglial cells. This has raised significant concerns about the roles of microglia in the pathogenesis of AD [24]. Studies have shown that microglia are susceptible to the pro-inflammatory effects of SP, which preferentially interacts with NK-1R on the surface of microglia to promote glial inflammatory immune response, thereby aggravating the damage of the CNS [25], and activating the following two major pro-inflammatory pathways [26].

#### 3.1.1 mTOR pathway

**Figure 2.**
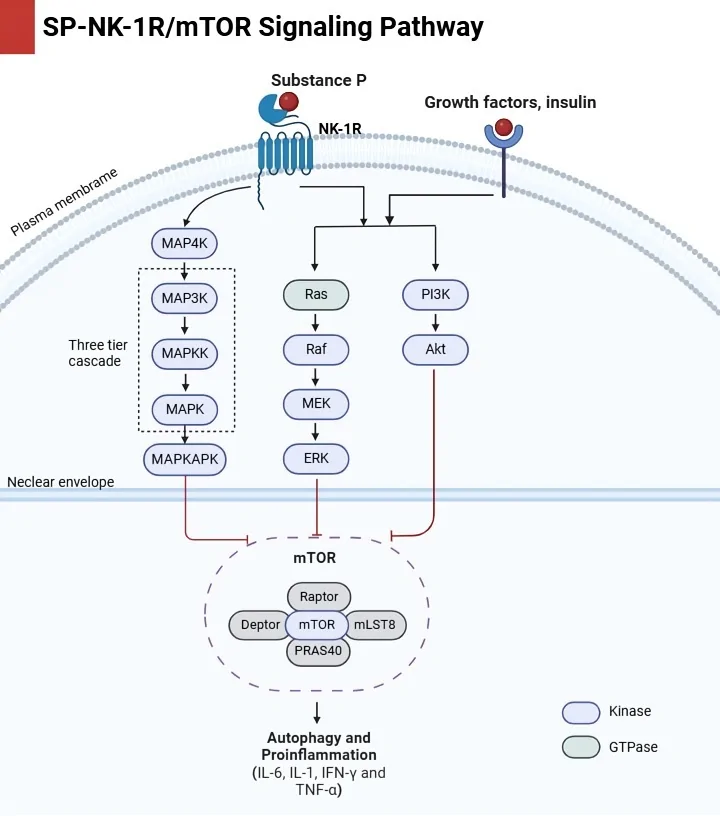
**SP-NK-1R/mTOR signaling pathway**. SP activation of NK1R on the membrane initiates G-protein-mediated signaling events, kinase-mediated ERK/MAPK signaling is sequentially activated by phosphorylation, and mitogen-activated protein kinase-activated protein kinases (MAPK-APK) and ERK1/2 can be transported to the nucleus to activate the important target mTOR leading to autophagy and proinflammation [27, 30]. In the traditional pathway, growth factors activate mTOR through ras/raf/MEK/ERK or PI3K/Akt pathways [27].

SP can activate the mitogen-activated protein kinase (MAPK)/extracellular signal-regulated kinase 1/2 (ERK1/2) cascade signaling pathway through NK-1R, which can further activate the important target, mTOR protein [27]. Notably, SP functions as the highest-affinity receptor for NK-1R and plays a significant role in activating ERK1/2, which is among the most crucial upstream signaling molecules involved in mTOR initiation. Some evidence also suggests that SP can act as a cytokine to activate the PI3K/Akt pathway; and PI3K/Akt, as an important pathway for activating mTOR, is also activated by growth factors, cytokines, immune activation, etc., to inhibit autophagy [28]. Consequently, SP emerges as a pivotal signaling molecule in the upstream regulation of the mTOR signaling pathway (Fig. 2) [27, 29].

##### 3.1.1.1 Autophagy

Autophagy is an important access to maintaining intracellular protein homeostasis. In the nervous system, neurons are highly differentiated and cannot dilute intracellular toxic or injured organelles through mitosis. Therefore, the role of the autophagic pathway in neuronal clearance becomes quite important [31].

However, the autophagic pathways are vulnerable during the process of neurodegeneration [31]. Previous evidence has found that there is a lot of accumulation of autophagosomes and autophago-lysosomes in the brain of patients with AD, which is thought to be due to the blocking of autophagy flux [32].

Additionally, the dysregulation of mTOR-mediated autophagy is strongly correlated with AD pathogenesis, a phenomenon that has been substantiated in both animal models and AD patients [33]. First, researchers have identified the accumulation of immature autophagic vacuoles (AVs) in AD brains, implying that under pathological conditions, autophagy fails to perform its clearance function, ultimately leading to the accumulation of Aβ. Previous studies have shown that apoptosis-related proteins (Bax, Caspase3 and caspase 9) decreased significantly in the hippocampus of rats treated with mTOR inhibitors. This research also found a higher number of autophagosomes and a lower expression level of overactive mTOR in the hippocampus of model rats from the intervention group. Additionally, it is also reported that the administration of mTOR inhibitors to AD model mice resulted in enhanced autophagy and a significant reduction in intracellular Aβ levels. Behavioral experiments demonstrated improved cognitive function in the experimental subjects [32]. Consequently, this suggests that mTOR can cause inhibition of autophagy, leading to neurological injury, while mTOR inhibitors can block this process.

Nonetheless, previous studies have failed to elucidate the precise mechanisms underlying autophagy dysregulation in pathological conditions, while the presence of SP suggests new perspectives for the study of this mechanism. The classical activation of mTOR is initiated by growth factors, such as insulin and insulin-like growth factors, which induce the activation of the PI3K/Akt or MAPK/Erk pathway. Ultimately, these 2 pathways activate mTOR through a series of downstream signal molecules [29]. Interestingly, SP can also act as the growth factor for mTOR activation [27], and is also presumed to activate downstream signal pathways and influence the autophagy regulation of mTOR. In other words, both growth factors and SP can convey high cellular energy signaling to mTOR, leading to the subsequent inhibition of autophagic activity [29, 31]. Consequently, after SP/NK1R triggered mTOR pathways which will lead to the inhibition of autophagy, SP may play a deleterious role in its potential mechanism to inhibit autophagy for disease progression.

##### 3.1.1.2 Proinflammation

Neuroinflammation refers to the inflammatory response of the CNS secondary to neuronal damage. Although inflammation in the body has a protective effect, the tissue damage and disease pathological progression can be contributed by an excessive inflammatory response [34]. A large number of studies have confirmed that SP plays an important role in enhancing the inflammatory response of myeloid cells such as macrophages and dendritic cells (DC) [35-37]. More and more studies have also found that SP can stimulate the microglia of the rat brain to produce IL-1, tumor necrosis factor-α (TNF-α) and other cytokines to aggravate the inflammation in the CNS [6, 38]. It is reported that SP can interact with NK-1R on the surface of microglia to activate the mTOR complex [6, 7]. SP activates NK-1R on the membrane of microglia to initiate G-protein-mediated signaling events. Kinase-mediated ERK/MAPK signals are sequentially activated by phosphorylation, and MAPK-APK and ERK1/2 can be transported to the nucleus to activate an important target, mTOR [27, 30]. This mTOR complex can influence the progression of AD pathology mainly in 2 ways: by regulating anabolic and catabolic processes such as autophagy, and by regulating the activity of microglia affecting the release of pro-inflammatory cytokines [39]. The interaction of SP with NK-1R triggers neurogenic inflammation and directly enhances inflammatory processes in the lungs, intestines, skin, and other peripheral organs [27]. Additionally, as the most abundant tachykinin in CNS, SP and NK-1R are predominantly expressed in non-neuronal cells, such as microglia, and play important roles in inflammatory response by activating mTOR through ras/raf/MEK/ERK or PI3K/Akt pathway. Similar to other peripheral organs, the interaction between SP and NK-1R has been shown to increase inflammation in the CNS, and these inflammatory reactions can accelerate the progression of AD [40]. Neurogenic inflammation is characterized by increased vasodilation and vascular permeability, leading to increased infiltration of immune cells, which promotes the release of pro-inflammatory factors such as tumor necrosis factor-α (TNF-α), interleukin-1β (IL-1β), interleukin-6 (IL-6) and interferon-γ (IFN-γ) [6, 7, 39]. The release of pro-inflammatory cytokines such as IL-6, IL-1, and TNF-α in microglia has intrigued researchers. Among these cytokines, mTOR activated by the interaction between SP and NK-1R on microglia induces neurogenic inflammation, which increases the expression of TNF-αand directly leads to the up-regulation of B-site APP lyase 1 (BACE1) expression [41]. As discussed in 1.1.1, within the amyloidogenic pathway, Aβ is dominantly produced by the continuous proteolytic cleavage of APP by β -secretase (also known as BACE1) and γ-secretase. As a result, up-regulation of BACE1 expression promotes cleavage of APP, which leads to increased production of Aβ, the core component of neurogenic plaques in the brains of AD patients [41]. Therefore, it is speculated that increased expression of TNF-α may exacerbate the disease progression of AD through BACE1.

Meanwhile, the pathological progression of CNS diseases such as AD is characterized by high levels of inflammatory mediators such as cytokines. Immunomodulatory neuropeptides, including SP, can promote the release of cytokines by interacting with NK-1R and activating the mTOR pathway [40]. In turn, studies have shown that inflammatory cytokines can activate the mTOR pathway through white blood cells, regulating the release of SP and the expression of NK-1R [35, 42]. Thus, a positive feedback loop between these cytokines and SP ultimately further promotes the progression of AD. In addition, SP activation of NK-1R on microglia can also promote the release of IL-6, IL-8 and IL-1β. These substances are responsible for blocking neuronal differentiation, attenuating microglial phagocytosis, and damaging the extracellular matrix by activating the accumulation of NF-κB and Aβ, ultimately exacerbating AD progression [43]. Both IL-1β and TNF-α increase the production of other inflammatory mediators such as prostaglandins and IL-6, suggesting that these cytokines work synergistically to regulate the pro-inflammatory cascade [44, 45]. Therefore, the increased levels of IL-1β and TNF-α will further aggravate the inflammatory response through the synergistic effect of cytokines, and thus promote the progression of AD.

Therefore, this proinflammatory mechanism started by the interaction of SP and NK-1R in the microglia, which activates mTOR to accelerate the release of proinflammatory cytokines, is the key to the pathology of AD and is related to its diagnosis, treatment and prognosis.

**Figure 3.**
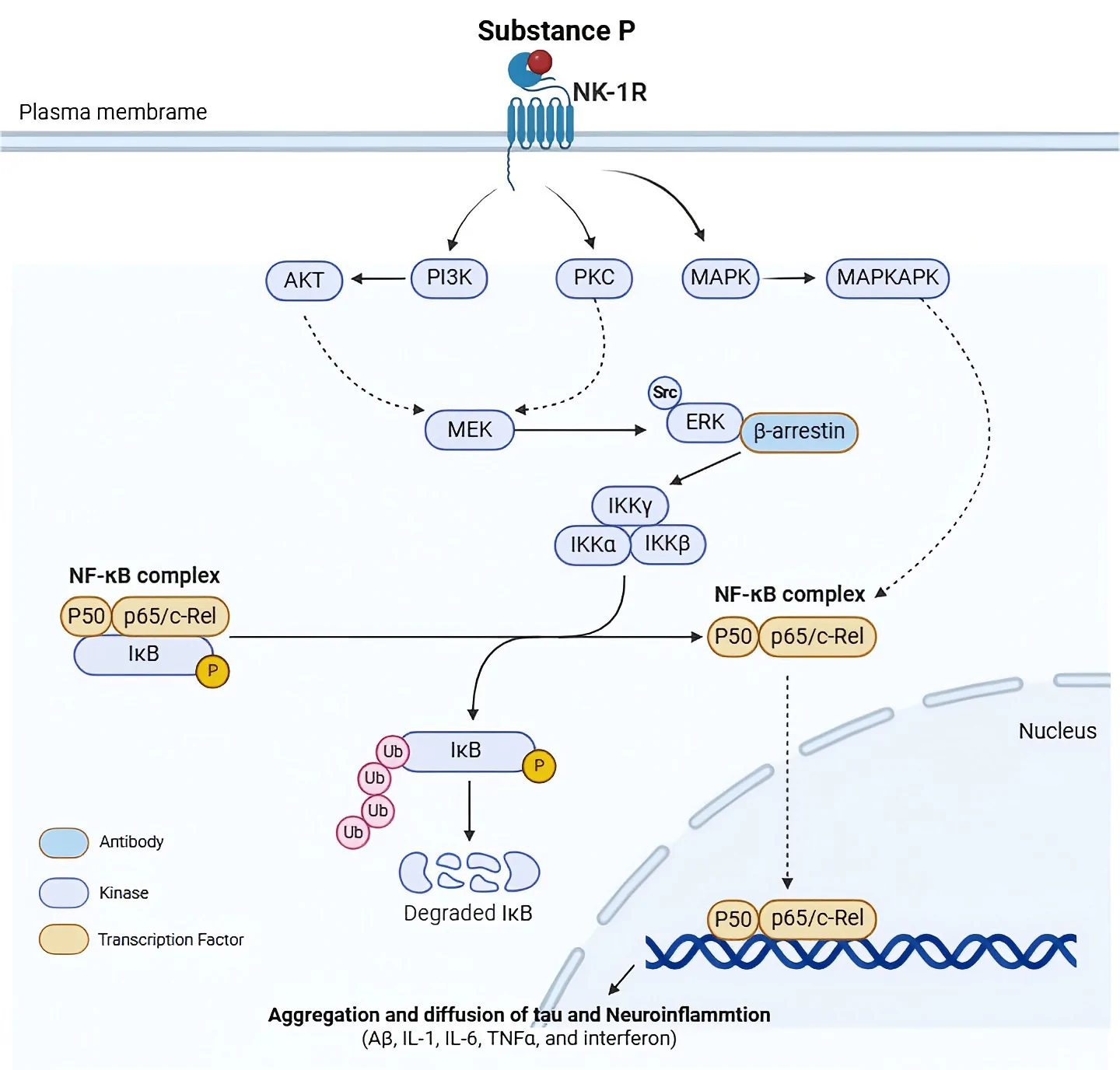
**SP-NK-1R/ NF-kB pathways**. SP binds to NK-1R to activate PKC or PI3K-Akt, which in turn activates MEK, leading to phosphorylation and activation of ERK1/2 and activation of NF-κB by inducing phosphorylation and degradation of the inhibitory protein IκB and nuclear translocation of its transcriptional active subunit p65 [2, 51]. At the same time, SP can also activate the MAPK cascade reaction and cause the activation of NF-kB, leading to aggregation and diffusion of tau and neuroinflammation [2, 50].

#### 3.1.2 NF-κB pathway

Research has demonstrated that the expression of nuclear factor kappa-B (NF-κB) is high throughout the brain and peripheral blood monocytes of AD patients [5]. In resting cells, the NF-κB p65:p50 dimer remains in an active complex. When neurons or glial cells are activated by various stimuli, such as the accumulation of Aβ, the p65:p50 dimer is released from the complex and transferred to the nucleus, which in turn induces the subsequent inflammatory response [46]. Examination of postmortem brain tissue from patients with AD revealed increased p65 immune reactivity in glial cells near Aβ deposits in AD brain sections, consistent with NF-κB activation in these cells. This suggests that NF-κB activity is increased in cells involved in neurodegenerative processes and is closely related to the pathological development of AD [47].

Studies have shown that the interaction between SP and NK-1R can initiate the activation of NF-κB, further activate the production of pro-inflammatory cytokines IL-1, IL-6, IL-12 and TNF-α, and aggravate the inflammation of the CNS [48, 49]. In the microglia, SP activates IκB kinase (IKK) leading to phosphorylation of IκB and subsequent release of NF-κB. At the same time, SP can also activate the MAPK cascades to cause NF-kB activation [2, 50, 51]. MAPK activation occurs through a three-layer phosphorylation cascade: MAPK is activated by MAPK kinase (MAPK or MEK), which is in turn activated by MAPKK kinase (MAP3K or MEKK) [52] (Fig. 3). Two activation pathways promote the release of pro-inflammatory cytokines.

Given the potential research value of NF-κB, it is currently considered a promising indicator of AD progression. This review will show the interaction between SP and NK-1R on microglia activates NF-κB signaling to exacerbate the progression of AD disease and discuss the following aspects: regulating pro-inflammatory cytokines and increasing Aβ accumulation, promoting AD-related neuroinflammation, and aggravating microglia-mediated tau proliferation and toxicity.

##### 3.1.2.1 Neuroinflammation

###### 3.1.2.1.1 Aβ

It is well known that Aβ is produced in large quantities and begins to accumulate in the brain during the development of AD [53], and the excessive production and accumulation of Aβ triggers a pathological cascade of AD, leading to neuronal cell dysfunction and ultimately brain cell death [54]. In addition, More and more studies found that increased SP levels in AD patients were positively correlated with Aβ_1-42_ levels [5]. Therefore, SP as an important promoting factor is closely related to Aβ accumulation to aggravate the pathogenesis of AD.

Multiple studies have reported that SP, which can initiate the activation of NF-κB, plays an essential part in upregulating diverse microRNAs in the brain, such as miR-146, miR-155, miR-181b, miR-21 and miR-301a [55]. Common to the aging, degraded brain and retina are significant upregulation of miRNA-125b and miRNA-146a, and their increase is positively correlated with AD progression (Lukiw, 2012). This upregulation leads to downregulation of regulatory proteins, such as tetraspanin-12, which impacts the activity of disintegrin and metalloproteinase-10, thereby transferring the βAPP processing activity to more amyloid and proinflammatory aβ production pathways [56].

It has been shown that Aβ production is not only a result of AD, but also acts as an upstream stimulus of NF-κB, which may be related to intracellular processes [54]. Activation of NF-κB by Aβ has been observed, and in return, it promotes the production of Aβ. Aβ accumulation could lead to damage of synapses, neuronal processes, and the blood-brain barrier (BBB), which subsequently leads to the infiltration of reactive microglia into the brain, and promotes the development of AD through positive feedback closed-loop effect [57].

As proposed by the amyloid cascade hypothesis, the core of senile plaques which are also called amyloid plaques are formed by accumulation of Aβ deposition in the grey matter of the brain, triggering the cascade that affects synapses and stimulates microglia activation, leading to neuroinflammation [58]. Additionally, It has been reported that the interaction of SP and NK-1R activates NF-κB, which promotes the increase of Aβ by inhibiting the cleavage of the α-secreting enzyme of APP, further accelerating the progression of AD [59]. This promotes the secretion of pro-inflammatory cytokines such as Pro-IL-1β, which induces neuroinflammation and promotes AD progression by activating the c-Jun N-terminal kinase (JNK), ERK, and NF-κB signaling pathways [60, 53].

Aβ deposition and neuroinflammation can interact with each other, and the SP-mediated NF-κB signaling pathway plays an important regulatory role in this process.

###### 3.1.2.1.2 Proinflammatory cytokines

Further studies have found that NF-κB can promote the progression of AD by inducing neurogenic inflammation [61], which is one of the main consequences of NF-κB activation. Additionally, it has been experimentally demonstrated that SP can initiate the activation of NF-κB in the microglia leading to an increase in proinflammatory cytokines, including IL-1, IL-6, TNFα, and interferon [62]. In addition, in neurodegenerative disease models, inhibition of microglial NF-κB delays microglial activation during AD disease progression and reduces inflammatory markers. At the same time, it has been proved that NF-κB activation is the mechanism of microglia-induced motor neuron death; while the inhibition of NF-κB can save neuronal death, prolong the survival time of experimental animals, and delay the disease progression [63].

Among these cytokines, SP promotes the activity of the NF-κB and ERK pathways and can activate the release of pro-inflammatory and cytotoxic factors, including TNF-α, IL-1β, IL-6, nitric oxide (NO), and reactive oxygen species (ROS). These mediators may directly induce neuronal apoptosis or amplify inflammatory responses, leading to synaptic dysfunction or neuronal loss, thereby driving the pathological progression of AD [64]. Additionally, Additionally, recent transcriptomic analyses of microglia models have shown that the upregulation of TNF-α is a significant contributor to the activation of NF-κB signaling. Studies have also confirmed that TNF-α can promote the aggregation of Aβ, confer neurotoxicity, and stimulate the production of various cytokines. The chronic increase of these cytokines has been shown to have neurotoxic effects [23].

The elevation of pro-inflammatory cytokines can, in turn, upregulate inducible nitric oxide synthase(iNOS) by activating NF-κB, leading to an increase in nitric oxide (NO), which can pose a threat to neurons, thereby exacerbating AD neuropathologically primarily through two mechanisms: (1) NO can promote the activation of NF-κB pathway by regulating neuronal migration during development, thus aggravating CNS damage and exacerbating AD disease progression [65]. (2) Excessive nitrogen peroxide and superoxide anion reactions will produce excessive peroxynitrite. Peroxynitrites have direct toxic effects, leading to lipid peroxidation, protein nitration, and DNA damage, thus exacerbating AD disease progression [66, 53]. These cytokines can also induce the expression of more cytokines and chemokines, leading to an inflammatory cycle [61]. This means there is also a positive feedback loop between NF-κB and proinflammatory cytokines. This upregulation of proinflammatory cytokines is considered one of the primary consequences of NF-κB activation [23].

SP can interact with NK-1R to activate the NF-κB pathway, promoting the production of pro-inflammatory factors, which are the basis of neuroinflammation and other pathophysiological processes, so this interaction is of great research value.

###### 3.1.2.2 Tau protein

NFTs containing highly phosphorylated tau are the pathological features of AD, and tau is the mediating factor of Aβ cytotoxicity, which promotes the neurodegenerative changes in AD disease [23]. NF-κB signaling in response to tau is the important cellular immune pathway in microglia [67]. That is to say, NF-κB activation is the key to accelerating the processing of tau protein in microglia, which accelerates the disease progression of AD mainly in two ways: by promoting tau hyperphosphorylation, and by activating tau propagation and seeding [23].

SP promotes the hyperphosphorylation of tau protein by activating NF-κB signaling. Additionally, studies have shown that NF-κB signaling can also be activated by tau in turn, exacerbating microglial-mediated tau propagation and toxicity [68]. Therefore, it is of great value to study the mechanism of the NF-κB activation initiated by SP for the treatment of AD. Recently, there has been increasing evidence that the activation of NF-κB by SP promotes tau hyperphosphorylation and plays an important role in increasing tau toxicity. Studies on this mechanism have shown that increased SP expression can lead to activation of NK-1R and increased activity of kinase including JNK, Akt, and ERK1/2, resulting in tau protein hyperphosphorylation. When tau is hyperphosphorylated, it remains in a separate state. On the one hand, the isolated hyperphosphorylated tau will destroy the nearby stability of microtubules and lead to synaptic degradation; on the other hand, it is also easier to aggregate, more likely to form NFTs, and ultimately accelerate AD progression [68]. Additionally, a series of studies have shown that SP also plays a role in inducing the expression of SET isoform 1 which is upregulated in the brains of AD patients, inhibiting the major tau phosphatase, protein phosphatase 2A, and thereby promoting the hyperphosphorylation of tau protein [69]. It contributes to the abnormal increase of cytoskeletal proteins, axonal transport disorders and neuronal degeneration, and aggregates to form neurofibrils, NFTs and other structures [58], causing cognitive and memory degradation and accelerating the progression of AD [70].

SP can activate NF-κB to promote tau propagation and seeding. SP activates NF-κB to promote microglia to secrete more tau with seeding ability, thereby accelerating the spread of tau protein [57]. It is reported that the effect of reducing tau seeding by inhibiting NF-κB signaling in microglia alone is similar to the effect of reducing tau seeding by complete depletion of microglia, which emphasizes the crucial effect of NF-κB pathway in mediating tau seeding in microglia [23]. Although the specific mechanisms by which NF-κB in microglia affects tau balance are not fully understood, alterations in NF-κB activity could modulate tau protein status which include exocytosis and/or proteolysis, which means the processes of tau releasing and/or proteolysis cleavage. Activation of NF-κB can promote microglia to secrete more tau protein with seeding capacity, thus accelerating the spread of tau protein pathology [23]. After the activation of the NF-κB initiated by SP, the activation of microglia is further increased by the progression of tau deposition, and thus the disease progression of AD is further aggravated.

Therefore, SP can activate NF-κB to promote the toxicity and spread of tau, which plays a crucial role in the progression of AD, but its underlying mechanism still needs to be further explored in detail, and it may become a new research hotspot with great research value.

### 3.2 Activation of mast cells

In SP-induced neuroinflammation, mast cells (MC) play a number of non-negligible roles. Previous studies have established that MC is one of the primary inducers of neuroinflammation [71] and activates signaling pathways in neuroinflammation that are distinct from microglia [72]. Recently it has been identified a significant role for MC in neuroinflammation and found that it is significantly regulated by SP [15].

MC is resident in CNS and is found mainly on the cerebral side of the blood-brain barrier (BBB) and distributed around glial cells when neuroinflammation occurs [15]. MC responds to SP stimulation mainly in an NK-1R-dependent pattern [6]. Upon stimulation by SP, MC degranulates and releases numerous types of cytokines, chemokines, and neuroactive substances, including IL-1β, IL-6, IL-8, IL-18, IL-33, TNF-α, vascular endothelial growth factor (VEGF), corticotropin-releasing hormone (CRH), CC motif ligand 2 (CCL2), dopamine, histamine, trypsin, prostaglandins (PGD) and many more. This range of substances also includes SP, implying a closed-loop positive feedback regulation between mast cell activation and SP.

Among the above mechanisms, there are several interesting links between SP and MC. SP can activate MC via NK-1R, and this activation in some neuronal diseases can contribute to the activation of NK-1R, and result in increasing vascular permeability [73, 74]. Another example is associated with cytokines, in which after MC is activated by SP, IL-33 will increase, cooperating with SP and MC in the inflammation processes. However, IL-33 can also improve the ability of SP to activate MC, resulting in generating a host of other cytokines [73].

As the main outcomes of the activation of MC, IL-1β and TNF-α can cause direct damage to neurons at high concentrations on the one hand, and glial cell activation on the other; at the same time, the IL-1 family can also positively regulate MCs, causing them to release more inflammatory mediators and amplify the neuroinflammatory response [15].

### 3.3 Activation of the HPA axis

In recent years, the role of the hypothalamic-pituitary-adrenal (HPA) axis in central nervous system disorders has been gaining attention. CRH activates the HPA axis, and it is proved to be the primary coordinator of the stress response. It has been found that SP upregulates corticotropin-releasing hormone receptor-1 (CRHR-1) on MCs, which can lead to increased production of IL-6, TNF-a and VEGF [75]. Also, IL-6 released by MC can promote CRH production by HPA axis activation [76]; and in neuroinflammation, CRH also promotes MC and glial cell proliferation [77].

That means besides directly regulating the HPA axis, SP can also modulate the HPA axis by MC. MC has been a most significant effector in the crosslink between the HPA axis and AD pathology. Firstly, as demonstrated above, MC can be activated by SP which can also upregulate the CRH receptors on MCs. This means MC will not only produce many cytokines which is positive feedback to itself, but also become more sensitive to CRH. Then, CRH will stimulate MC, making it degranulate and release an array of cytokines [78]. Of note, the degranulation of MC can release histamines and CRH, which also forms a positive feedback loop. Early in 2001, it was reported that brain-resident MCs can act as an immune gate by modulating the HPA axis through this process.

Several studies in recent years have also reported the involvement of CRH in the pathology of AD, including the promotion of Aβ production [79, 80], indicating its importance in AD.

The role of the HPA axis that plays in AD is of great significance. In the early stage of AD, dysregulation of the HPA axis has been observed, associated with the detection of elevated CRH levels in patients’ body fluids [78]. It has also been reported that brain glucose metabolism and CRH level have shown a significant association, indicating the unneglectable role of the HPA axis in AD. Evidence also showed that CRH can regulate the activity of γ-secretase, thus increasing the level of Aβ. Animal experiments even made some nice attempts to discover the potential drug targets according to the HPA axis. The phosphorylation of tau was inhibited in mice with CRHR1 (there are 2 types of CRH receptors, CRHR1 and CRHR2) knocked out, while the CRHR2 knocked ones didn’t show the similar phenomenon. This means CRHR1 mediated a special pathway in AD pathology, which can probably be turned into a new drug target. And the CRHR1 antagonist has shown a promising future, as one antagonist R121919 has been proven to be able to eliminate the deposits of Aβ and suppress the level of phosphorylated tau to some extent. Thus, as discussed above, SP, the upstream regulator of the HPA axis, is also indispensable in AD pathology.

### 3.4 Acting on immune cells

The regulatory effects of SP on immune cells are exerted in the following three aspects: i) regulating the activation of immune cells, promoting their survival, proliferation and chemotactic migration; ii) indirectly regulating the immune function of leukocytes by regulating various cytokines and other active mediators; iii) activating adaptive immunity and stimulating the secretion of immunoglobulins.

#### 3.4.1 Regulation of immune cells

First of all, in general, SP can enhance neutrophil phagocytosis by inducing respiratory bursts and increasing the production of reactive oxygen intermediates [81]. SP can also enhance the chemotaxis of neutrophils through increasing the expression of IL-2 [80].

For the human natural killer cells (NK), SP can enhance the cytotoxicity of NK cells, upregulating the production of NK cell-related molecules, which includes perforin, granzyme, and natural cytotoxicity receptors [82].

For T cells, SP can stimulate T cell proliferation through increasing the expression of IL-2 [81]. Studies also demonstrated that NK-1R-negative mice showed suppressed T cell proliferation, suggesting that SP-NK-1R signaling plays an important role in T cell proliferation [81]. SP can influence the migration of T cells as well. In activated T cells, SP can enhance their expression of macrophage inflammatory protein-1β (MIP-1β), which is basically a chemokine that can manipulate T cells to move toward the inflammatory regions [81]. This procedure is regulated through NF-kB, and can be interrupted if NK-1R antagonist CP-96,345 is given [81]. Patients with AD are clinically able to be observed an imbalance in peripheral T-cell subsets, usually with a decrease in CD8+ cells and an increase in CD4+ cells [15]. Therefore, nowadays, T-cell subpopulation imbalance is hypothesized as a part of AD pathology. Whereas it has been reported that after administration of SP, the secondary immune response could be amplified by activating CD8^+^ T lymphocytes during the primary immune response. SP also biases the inflammatory response towards Th17 immunity and modulates the Th1/Th2 balance towards a Th1 or Th2 response depending on the nature of antigens [81]. SP can increase the production of human memory Th17 cells by inducing monocytes to express IL-1β, IL-23 and TNF-like ligand 1A [83]. To turn CD4+ memory T cells into Th17 cells, there is another alternative, which is that SP can upregulate CD4+ memory T cells’ expressions of IL-17A and IFN-γ, turning non-Th17-committed CD4+ memory T cells into real Th17 cells and Th1/Th17 cells [83]. And this process is also mediated by NK-1R. Furthermore, by binding to NK1R on DCs’ membrane, SP can induce a Th1 bias in mouse effector T cells [81]. Th1 and Th17 cells are pivotal in the pathology of many inflammatory diseases, therefore understanding the factors that regulate T cell subpopulation differentiation is essential for future directions of chronic inflammatory disease treatment.

For DCs, SP can modulate the chemotaxis of DCs towards lymph nodes by regulating chemokine receptors and adhesion molecules. For instance, SP upregulates the expression of the chemokine receptor CCR7, as well as the expression of the adhesion molecule Mac-1 antigen (CD11b/CD18) and its ligand ICAM-1 (CD54) on NK-1R positive DCs, which facilitate these cells moving towards lymph nodes [84]. Meanwhile, SP can also improve the survival of DCs, which is manipulated via NK-1R [85]. Intriguingly, no matter what stage is DC, they will continually express NK-1R; and when under inflammatory stimuli, DC will upregulate its expression of NK-1R mRNA, meaning that inflammation can stimulate DC to upregulate NK-1R. These processes form an interactive relationship between DC and NK-1R, which can be activated by SP in inflammatory conditions [85].

Moreover, SP can also potentially determine the endothelial-leukocyte interactions by inducing the expression of an endothelial-leukocyte adhesion molecule in the microvascular endothelium [81]. The changes in endothelial-leukocyte interactions would exert an enormous impact on the BBB, which is a significant part of AD-relevant neuroinflammation.

#### 3.4.2 Regulation of related cytokines

The association of proinflammatory cytokines and AD symptoms has been presented in many clinical studies. It is reported that cognitive decline for 6 months has synchronized with the increase of proinflammatory cytokines [86]. Furthermore, compared with low baseline TNFα levels, the higher baseline TNFα levels have presented a correlation with faster cognitive decline, suggesting that cytokines play an unneglectable part in AD clinical symptoms [86].

And SP has shown a great capacity of regulating a variety of immune cells to generate a host of various cytokines [81]. As described above, SP can modulate MCs, HPA axis, T cells and DCs, which can all produce cytokines and use cytokines as major effectors to exert further impacts on other pathophysiological processes. Among these cytokines, the effects they can bring are also of great diversity. For instance, IL-1 is pro-inflammatory; IL-8 has chemotactic properties, while IL-10 can modulate immune functions [81].

However, intriguingly, the regulation of leukocyte-associated cytokines by SP is a bidirectional mechanism, with SP regulating the release of cytokines, which in return regulate the effects of SP. For example, IL-12, IL-18 and TNFα can induce the expression of NK1R in T cells, while IL-10 and TGF-β inhibit the activation of NK-1R. Meanwhile, not only SP can activate leukocytes, but many leukocytes are also able to express SP in large numbers [81].

#### 3.4.3 Activating adaptive immunity

SP can participate in the activation of adaptive immune cells. It is reported that SP enhances the secretion of immunoglobulin from Peyer's patches, splenic lymphocytes and mesenteric lymph nodes in mice in an isotype-specific manner [81], confirming the critical nature of SP in immune regulation. In the meantime, if stimulated with lipopolysaccharide, SP would increase IgM secretion 3 times, which also depicted that NK-1R and Toll-like receptor 4 (a receptor which can activate B cells to secrete IgM) have a crosstalk [81].

As discussed in 2.3, SP has a significant role in the HPA axis regulation. Whereas, the HPA axis can not only directly influence AD pathology, but also influence the ontogeny of adaptive immunity [87]. The influence of the HPA axis mainly lies in regulating the functions of thymus, which is central to T cell generation and immunological homeostasis modulation. Early in 1936, studies showed that the activation of the HPA axis can lead to the involution of the thymus, which was bound to affect the subsequent T cell-involved immunological processes [87]. According to the former discussion in 2.4.1, T cells play a significant part in AD inflammatory pathology, suggesting that the influence made by the HPA axis and SP is also important in AD.

### 3.5 Increasing blood-brain barrier permeability

The permeability of the BBB is increased during systemic inflammation, which is a vital part of the pathogenesis of AD [88]. Under physiological conditions, the BBB restricts the passage of macromolecules and peripheral cells from entering the brain. However, during systemic inflammation, peripheral immune and inflammatory mediators can act on endothelial cells in the BBB, inducing more production of the inflammatory mediators, such as PGD2, and disrupting the BBB [74]. In recent years, it has been found that increased BBB permeability may induce neurodegeneration [89]. Mounting evidence has been found to elucidate the relationships between BBB and abnormal protein accumulation in neurodegeneration, vascular damage and inflammations [90]. Infiltration of inflammatory mediators and immune cells further disrupts the BBB, leading to a further increase in its permeability, and creating positive feedback.

The effect of SP on BBB permeability is not limited to the broad outcome triggered by neuroinflammation, but has a more specific effect. SP not only induces NO production by endothelial cells and thus directly regulates angiogenesis [91], but also enhances BBB permeability by disrupting tight junction proteins [92]. SP has also been found to indirectly regulate angiogenesis through its interaction with MC and granulocytes; it can promote MC expression of VEGF; and after SP activation of MC, its degranulation produces IL-33 which also enhances MC expression of VEGF [93]. There is also therapeutic evidence in this regard, with studies claiming that treatment with NK-1R antagonists is beneficial in defending the integrality of the BBB and may slow the progression of certain neurodegenerative diseases or prevent some early neurodegenerative dysfunction [94]. A new study in 2023 has recently proved that SP can reversibly compromise the integrity and function of BBB via NK-1R activation, with the activation of RhoA/ROCK/MLC2 pathway, NO and ERK1/2 also observed, which may be the underlying mechanisms [95]. This new study further depicts the unneglectable role of SP in BBB regulation, which is also an unneglectable part of AD.

## 4. Discussion

### 4.1 Neurokinin 1 receptor isoforms

NK-1R, a receptor that mainly exists in glial cells such as microglia, has the highest affinity for SP among all the other neuropeptide receptors and is currently the best receptor for the study of SP. NK-1R is widely expressed in non-neural cells of CNS (e.g., astrocytes and microglia) which have lots of important immune functions and are involved in many CNS physiopathological processes, including neurodegenerative diseases such as AD (Ek et al., 2001).

NK-1R, as a G protein-coupled receptor, contains two isoforms: full-length type and truncated type. They contain different numbers of amino acids, as well as different lengths of carboxyl termini of amino acids. Particularly, when SP interacts with full-length NK-1R and truncated NK-1R respectively, the biological reactions mediated by these two subtypes exhibit notable differences [11].

**Figure 4.**
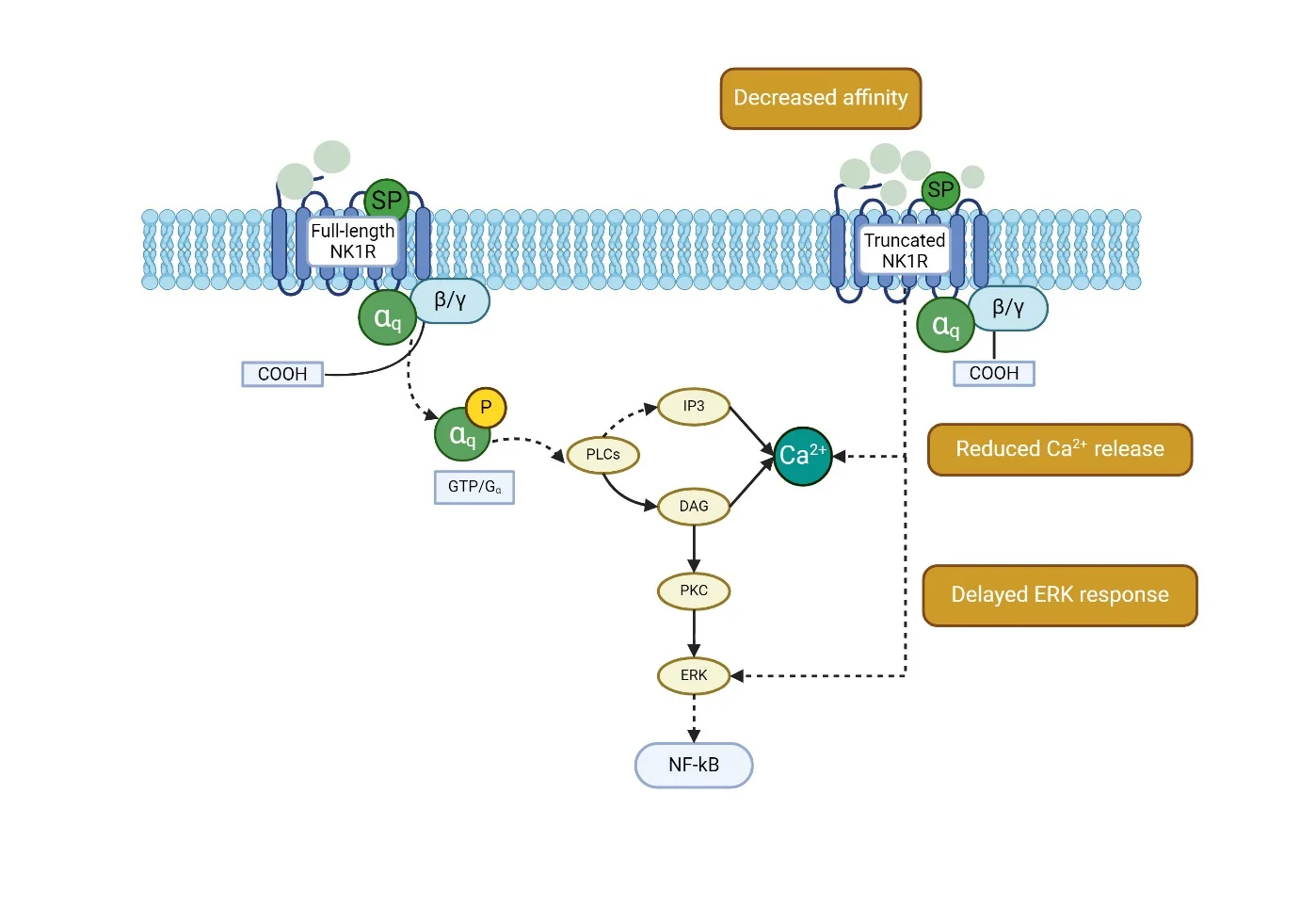
**Two isoforms of NK1R**. SP acting on full-length NK-1R can lead to elevated calcium levels and the activation of NF-ĸB. In contrast, SP acting on truncated NK-1R leads to reduced calcium levels released, delayed ERK response and reduced NF-ĸB activity relatively [96]. Furthermore, the absence of the C-terminal of truncated NK1R impures the internalization and recycling of the receptor, and ultimately the affinity of SP to truncated NK1R is reduced [96, 97].

It was shown that in cells expressing full-length NK-1R, SP can lead to elevated calcium levels, which are required for NF-ĸB activation, and that this pathway can ultimately lead to increased NF-ĸB activation [11]. In contrast, SP acting on truncated NK-1R fails to increase intracellular calcium levels and thus cannot activate NF-ĸB [96]. At the same time, although the truncated type can also activate ERK (a kind of extracellular signal-regulated kinases, which can be activated by SP to activated downstream signaling pathways) relative to the full-length type, the slow rate and the different timing of ERK activation also led to the effect of truncated NK-1R in reducing NF-ĸB activity. That is to say, the full-length NK1R can activate NF-ĸB, while the truncated NK1R cannot [96] (Fig. 4).

Although the distribution of different NK-1R isoforms on the cell membrane is still not clearly investigated, full-length and truncated NK-1Rs may provide one aspect to explain the double-edged effect of SP:

(1)The coexistence of truncated and full-length NK-1R receptors on the cell membrane may produce competing effects, which may lead to two different outcomes of SP protection or damage to AD due to a balancing process. However, so far there has been a lack of relevant evidence to firmly support this hypothesis. More research is needed in the future.

(2)It is shown that almost all NK-1R expressed in microglia are full-length type, and full-length NK-1R is prolifically expressed in CNS [11, 97]. Since neurons damaged by excessive inflammation are unable to regenerate, NK-1R receptor-mediated excessive inflammatory responses often pose a toxic threat in the late stage of AD. Therefore, blocking the full-length NK1R, which is the majority in CNS, may block most of the inflammatory response while still preserving the rest of the immune function served by the truncated form. Specifically blocking or antagonizing the full-length forms may greatly block the neurotoxicity associated with an excessive inflammatory response. That is particularly beneficial for patients with late-staged AD where neuroinflammation has cascaded and exerts a totally deleterious effect.

In recent years, the function of NK1R isoforms, especially truncated NK1R (NK1R-T), has not been widely studied. NK1R is of great significance in various complex brain functions, including neuronal sensory transmission related to emesis, pain, depression, anxiety and central responses to stress. NK1R antagonists (such as aprepitant) are currently widely used to suppress vomiting caused by chemotherapy drugs [98]. However, some evidence has been demonstrated that NK1R-T distributed on monocytes has an effect on immune regulation. SP via NK1R-T does not lead to the mobilization of calcium ions to activate NF-κB, but Chernova et al. have reported that SP can enhance Ca2+ mobilization stimulated by CCR5 ligand CCL5, leading to chemotaxis [81]. However, it remains unclear how the two subtypes of NK1R function under pathological conditions. The properties of different NK1R isoforms need further study and should be considered when devising therapeutic approaches targeting NK1R signaling [98].

### 4.2 Stress and anxiety factors

Multiple studies have provided evidence regarding the involvement of SP in modulating stress and anxiety responses. Endogenous SP has been found to be released in specific brain regions, commonly associated with stress and anxiety mechanisms, such as the amygdala and vomeronasal nucleus, in response to stressors. Thus, the responses to stress and anxiety are thought to be associated with high levels of SP immunoreactivity in the central nervous system [99].

In contrast, the HPA axis is the main system responsible for regulating physiological and behavioral patterns in response to physical and psychological stressors in vivo. The modulatory role of SP on the HPA axis through MC has been described previously. According to studies, both the degranulation of MCs and the activation of the HPA axis can be upregulated when under pressure [78]. As discussed before, SP, MCs and the HPA axis have a series of crosstalk, meaning that stress and anxiety may affect their activities in a complicated way. It is hypothesized that whether or not an AD patient suffers from stress and anxiety may influence the expression of their endogenous SP, which exerts regulation on the course of AD through the HPA axis, thus explaining the diversity of SP levels in AD patients [5].

However, stress is a more complicated factor in AD that can bring much more diversity than the above paragraph expected. As AD is a disease that is more popular in the aging population, aging is also an important factor that needs to be considered as a significant characteristic that most patients possess. Studies have reported that in aging populations, microglia are more sensitive to stress, which may increase the vulnerability of aging patients to neurodegeneration and decline in their regenerative capacities [16].

This reveals the complexity of AD pathology. It is not only under a lot of complex regulation, but the major patient group also has their particularity, for instance, aging. And the particularity can affect the performances of many regulating factors, as discussed above. However, the influence level of aging varies in individuals, which may partially explain the complexity of the clinical manifestation of SP and AD. This also calls for more rigorous experimental research to discuss the potential variants in SP regulation and AD pathology more comprehensively.

### 4.3 SP levels

At present, there are only very few studies on the change patterns of SP levels in AD patients. It is still not very clear how SP level fluctuates in different AD stages and in different tissues, revealing some contradictions and individual differences.

According to previous studies, the SP level increases in the following conditions: Johansson found that CSF SP levels rise in patients with early-onset AD (EOAD) [5]. In EOAD, neuropeptidases that break down SP, such as neutral endopeptidase E.C.3.4.24.11 and metalloendopeptidase E.C.3.4.24.15 are disrupted [100], leading to an extended metabolic half-life of SP in the temporal cortex of patients with senile dementia, which causes the increase of SP level [101]. Meanwhile, Rösler also detected a notable elevation of SP in patients diagnosed with late-onset AD (LOAD) [102]. In Ahmed’s experiment, SP level increased in the pallidum and substantia nigra, suggesting the fluctuation of SP may have a specificity of brain regions [103]. A recent animal study showed that in AD mice models constructed with Aβ1-42 injection, SP levels were significantly upregulated in the hippocampus and frontal cortex, indicating that SP levels may dominantly react to Aβ1-42 environment, particularly in the hippocampus and frontal cortex [104]. Johansson’s experiments showed similar results that SP has been found to have a positive correlation with the level of Aβ1-42, which serves as a biomarker for the disease status in AD patients [5]. Thus, we assume that elevated SP levels in CSF may play a compensatory role in protecting patients from pathological exposure.

Meanwhile, SP level decreases in the following conditions: SP level is different in different brain areas. Decreased SP levels are seen in the cortex, hippocampus and striatum [103]. It also differs across different stages of AD and tissues. A recent review indicated that EOAD reduces SP levels in several brain regions, including the hippocampus and striatum [101], while in Johansson’s study, a decrease in SP levels has been observed in the cortex of the brain and CSF in some post-mortem AD patients (Johansson et al., 2015)[5].

However, because the existing studies have not controlled all these confounding factors precisely, and the relationships between AD and the above factors are still not clear. For example, in the study by Johansson et al. in 2015, it was shown that the levels of SP in CSF of AD patients increased, which is controversial compared to previous experimental results. There are some limitations including a small sample size of AD patients (n=32), which could make the outcomes potentially influenced by chance and could potentially affect the representativeness of the data. Additionally, the inclusion of various dementia types in the "other dementia" group and inconsistent follow-up durations (ranging from 1 to 7 years) may confound interpretations of disease progression. Moreover, the lack of long-term follow-up data limits insights into the natural history and prognosis of the diseases under study. We consider that this may indicate that the clinical research of SP still needs improvement (Table 2).

**Table 2:** SP in AD: The summary

SP levels	Types of AD	Species	Brain Regions	Conclusion	Research
**Increase**	EOAD	Human	Temporal cortex	EOAD disrupts neuropeptidases (E.C. 3.4.24.11 and E.C. 3.4.24.15), extending SP's half-life thus increasing SP levels.	Gurram et al., 2024
	Unknown	Mice	Frontal cortex	In AD models injected with Aβ 1-42, SP levels significantly increased in the hippocampus and frontal cortex of mice.	Satarker et al., 2024
	EOAD	Human	CSF		Johansson et al., 2015
	LOAD	Human	Unknown		Rösler et al., 2001
	Unknown	Human	Pallidum & Substantial Nigra		Ahmed et al., 2004
	Unknown	Human	CSF	SP has been found to have a positive correlation with the level of Aβ1-42.	Johansson et al., 2015
**Decrease**	Post-morterm AD patients	Human	Cortex of brain & CSF		Johansson et al., 2015
	EOAD	Human	Hippocampus & Striatum		Gurram et al., 2024
	Unknown	Human	Cortex, Hippocampus & Striatum		Ahmed et al., 2004

SP, substance P; AD, Alzheimer’s disease; EOAD, early onset Alzheimer’s disease; LOAD, late onset Alzheimer’s disease; Aβ, amyloid β; CSF, cerebrospinal fluid.

## 5. Guesses of the two-sided effects of SP

In different stages of AD, the expression of SP is markedly different in CSF and other tissues. Johansson has mentioned that in the progression of AD, SP concentration in CSF increases in the early stage of AD [5], while in another study, CSF SP level was decreased in late AD (mean age 87 years) but not in younger AD patients [105].

Previous research has demonstrated that among individuals diagnosed with AD, specifically those who have senile dementia, there is a reduction in the activity of neuropeptidases responsible for breaking down SP in the temporal cortex. Consequently, this leads to an increased duration of SP's metabolic process in the temporal regions [106]. Meanwhile, it is worth noting that the most extensively validated imaging indicators in AD include medial temporal lobe atrophy observed through MRI, cortical Aβ deposition visualized with amyloid-PET imaging, and posterior cingulate and temporoparietal hypometabolism detected by 18FDG-PET. Among these biomarkers, two are specifically related to the temporal region [107], indicating that the temporal region has a strong correlation with AD, thus the distribution of SP may also have meaning to AD pathology.

Another significant implication of the SP study is its detectability in blood, offering a minimally invasive alternative to the previously used CSF detection method. This advancement significantly reduces patient discomfort associated with invasive procedures, thereby underscoring the importance of SP research.

Moreover, SP can alter the neuro-immune status of the brain. SP can normally activate the neuroinflammatory response, which is a defense mechanism of the body against stimuli. In neurodegenerative diseases, neuroinflammation can help eliminate infections in the early stage and, to some extent, promote the repair and regeneration of damaged neurons. However, SP can also activate microglia, mast cells and other immune cells, leading to the deterioration of neuroinflammation. SP may alter microglia and other immune cells, from the stable status or the homeostasis entering into the disease-related mode, which is considered as a canonical part of AD pathological progression.

Combined with the facts above, we speculate that in the early stage of AD, in order to eliminate disease states, the body secreted SP to protect the nervous system. For example, SP can activate ADAM 9, increase sAPPα secretion and protect neurons from apoptosis [10], in the meantime, SP can downregulate Kv4 channel expression to protect neurons from apoptosis induced by Aβ [108]. As mentioned above, SP can also co-aggregate with Aβ to reduce its toxicity and produce neuroprotective effects [14]. However, with the progression of AD, overproduced SP may decompensate the central nervous system, aggravating AD by activating microglial cells, mast cells, white blood cells, and other pathways, which can damage the CNS. We describe the double-edged effect of SP as the regulatory network of SP towards the nervous system. Considering such plenty of complex pathways, the associations and interactions of different mechanisms still need further investigation.

The elucidation and in-depth study of this mechanism may become an important basis for delineating the therapeutic stages of AD, and the related biological processes may also provide new drug targets and therapeutic ideas for AD.
